# A Case of Recurrent Paralysis of the Motor Oculi

**Published:** 1887-12

**Authors:** O. F. Wadsworth


### A CASE OF RECURRENT PARALYSIS OF THE MOTOR OCULI.

BY O. F. WADSWORTH, M.D.

The case which I wish to report appears to belong to a class characterized by a very distinctly defined group of symptoms. At intervals varying in length both in the individual cases and the same case, there occurs paralysis of all the branches of one oculo-motor nerve. The duration of the paralysis also varies. Always it is the same nerve that is affected, the other nerves remaining free. In most cases, possibly in all, the attacks are preceded, or, at least, accompanied by headache, confined to the corresponding side of the head, and chiefly localized in the frontal region.

Pauline and Frances, twins, were born of healthy parents, both of whom are still living and well. There were five other children in the family—two older, three younger than the twins. With the exception of a girl, the second after the twins, who died at eleven months of "congestion of the brain," all were healthy. In June, 1874, when the twins were nearly three years old, both had scarlet fever, and both had a discharge from the ear. Both seemed to recover well and to be healthy for some time afterward, although in both a slight discharge from one ear continued, and Pauline had occasional headaches.

Although Frances is not the one whose case I present, it may be of interest to follow briefly her history, as it seems to have a bearing on the etiology of the other case. In September, 1877, when six years old, three years after the attack of scarlet fever, she began to complain of headache. The headache returned more frequently, and was of longer duration till the following December, when she had a convulsion. Three weeks later convulsions recurred, and the attacks became more frequent until, finally, they occurred almost daily. About the middle of March, 1878, after a convulsion, she complained that she could not see. A week latter, March 21st, she was admitted to the nervous department of the City Hospital. On the same day I found well-marked optic neuritis and many white spots around the macula in both eyes. May 13th, Dr. J. Orne Green found the left membrana tympani much thickened and drawn in. She remained in hospital eleven weeks. The headaches and convulsions ceased, and the discs became atrophic. Sight did not return. I saw her again February 24, 1879, in the out-patient department. She had had an occasional headache since leaving the hospital; but was otherwise, excepting her blindness, well, well-nourished, of good development, rosy-cheeked. She could only distinguish light from darkness. The pupils were large; the optic discs presented the appearance of atrophy following neuritis. Since that time I learned that she has been well, free from headache, and has had no discharge from the ear. She is now a pupil at the Perkins Institution for the Blind, in South Boston.

Pauline, the subject of the oculo-motor paralysis, after the scarlatina in the summer of 1874, had attacks of headache every month or two ; in the early part of 1878, headache for several hours each day during three weeks. The monthly or bi-monthly headaches continued, and, on February 24, 1879, being then seven years old, she was brought to the hospital, with her sister, having suffered from headache daily for two weeks. The headache was referred to the right supra-orbital region; was said to commence every day about noon, to intermit about three, and to recur about five, and then continue some four hours. There was vomiting on the first two days. Appetite impaired; dejections and micturition normal; tongue clean; general appearance good; a slight discharge from right ear; swollen glands behind each ascending ramus of jaw. Dr. Green examined the ears, but my notes state only that he found inspissated pus in the right. The right eye was quite normal, its refraction nearly emmetropic; the left eye highly myopic, with a regular crescent of one-third to one-half the diameter of the disc.

March 29, 1880, she was seen again. Meanwhile, headache every month or two, usually ushered in with vomiting, and lasting from one to three days. When free from these attacks, she had appeared perfectly well. She was at this time suffering from headache which had lasted a week, the pain referred, as always, to the right supra-orbital region. There was vomiting at the beginning of the attack. She moaned in her sleep. Tongue clean; dejections regular; pulse 100, temperature 98.8°. Five or six days after this attack began, ptosis and divergence of the right eye was noticed.

There was paralysis of the right motor oculi. In other respects, the condition of the eyes as before. Dr. Green again examined the ears, but my notes only describe the condition he found in the left. Its membrana tympani was much drawn inward and opaque from swelling, and there were several spots of ecchymosis. Its hearing was very fair.

At the end of Feburary, 1887, Pauline came again to the hospital. She was now fifteen, well grown, and apparently healthy. Her headaches had diminished in frequency; for the last few years, she had had three or four each year, lasting from one day to two weeks, the more severe ones being accompanied by complete ptosis of the right eye and dilatation of the pupil. She had noticed no diplopia at these times, and no special change in the position of the eyeball. She stated that the ptosis and dilated pupil always continued till the headache was over, and then gradually passed off, so that at the end of a week or ten days they had entirely disappeared. The latter statement was probably incorrect, and I shall refer to it again. She had just recovered from a headache of two weeks' duration, accompanied by vomiting. There was a moderate amount of ptosis of the right eye, and nearly complete paralysis of all the other muscles, external and internal, innervated by the right motor oculi. Moderate divergence. The external rectus and superior oblique acted normally. Fundus of both eyes as before. She was seen again twice, at intervals of two or three days.

March 4th, there was a little more movement of the eye inward, upward, and downward; a little more action of the pupil and of the ciliary muscle. R., V. $=\frac{25}{20}$. L., with —14 D., V. $=\frac{20}{70}$. Field normal. She promised to come again, but did not.

April 24th, I visited her at her home. There was still a slight droop of the right upper eyelid, decided impairment of movement of eye upward, moderate impairment of movement downward and inward. The pupil did not react fully, and power of accommodation was imperfect for her age. She was, however, conscious of no defect of motion in the eye, nor aware that the pupil was affected. This was eight weeks after cessation of the headache, and it is probable, therefore, that previous paralyses had not passed off completely, as she had stated, especially as her father had observed that her eyes usually appeared not quite straight. That she should never have been troubled by diplopia is not surprising in consideration of the great myopia and imperfect vision of the other eye.

I was unable to see the mother, who was away at the time of my visit; but the father was positive that the attacks had diminished in frequency of late years—the last severe one having been in the previous July, an interval of seven months. Another fact of much interest I first learned from him, that with each severe attack, toward the end of the period of headache, there was a free discharge of ill-smelling fluid from the right ear, staining the pillow, and requiring syringing for some days. At my request and representation of the importance of treatment of the ear, she visited Dr. Green on May 6th. He found a large polypus completely filling the right meatus and removed it, but after two or three visits she ceased attendance.

These cases apparently occur but rarely. I have found but fifteen reported, with one exception already collected by Mauthner, although very possibly some publications during the last year have escaped my search. And of these fifteen several are very meagerly reported—two in such a way that it seems by no means certain that they properly belong to this category. On the other hand, with one exception, reported by Gubler in 1860 (Mauthner has recently published notes of a case seen in Von Graefe's clinic in 1864), the earliest observation published was in 1882, so that it is probable that the number of reported cases will increase considerably in the immediate future.

The sex is not mentioned in one of the doubtful cases; of the remaining fifteen, including mine, four were males, eleven females.

In the two cases I have regarded as doubtful the side is not given ; of the other fourteen, in seven the right, in seven the left motor oculi was affected.

The age at which the paralysis first appeared is not given in three cases, including the two doubtful ones; in one it occurred at eleven months, in one at eighteen months; the latest date, with one exception, is fourteen or fifteen years. The exception is a case briefly referred to by Buzzard, a woman of thirty, " subject for years to paroxysms of facial tic, recurring about every fortnight, and concentrated more or less definitely in the ophthalmic division of the fifth nerve." Within the last year or two each attack had been followed by partial paralysis of the oculo-motor, the paralytic condition lasting for a few days. One of the attacks he observed.

The varying length of the intervals between the attacks has already been noticed. In the case just cited it was shortest—two weeks. In Von Hasner's case the attacks recurred every month, and although preceding by two years the establishment of the catamenia, after their appearance were coincident with them. In most cases the interval was not a regular one, and lasted from a few months to a year. In Gubler's case there were two intervals of three years, then one of sixteen years before the last attack, which was followed a fortnight after its commencement by death.

The period of headache varied, even in the same patient, in one case from one day to several weeks. And a simiar difference appeared in the duration of the paralysis. Although the patients often are said to have stated, as in my case, that the paralysis passed off completely in the intervals, there is, I believe, in no case the definite statement that its entire disappearance was observed by the reporter. On the other hand, in several instances evident remains of the paralysis were observed to persist between the attacks.

With four exceptions, it is distinctly stated that pain resembling megrim accompanied the attacks. Of the exceptions, Gubler's patient was seen only some days after the paralysis was established; the case is reported very briefly, but it is said that the man had all his life been subject to violent attacks of megrim. Weiss's case is also very briefly reported (the patient was of little intelligence and unfamiliar with the German language); nothing is said of pain. The other two are the cases classed as doubtful. These were reported in a discussion which followed a report of a case by Snell. In one, by Ormerod, it is not clear that there were ever more than two attacks; the patient was an elderly woman; the Argyll Robertson symptom was present, but the knee-jerk not wanting. It is stated that there was pain in the region of the supra-orbital nerve, but nothing to suggest megrim. Of the other, by Beevor, we learn only that he "had seen a similar instance in which exposure to cold winds were (*sic*) assigned as the cause." Perhaps both these cases had better have been omitted from the list.

Only in one case, that of Thomsen, was there observed contraction of the field of vision during the attack; and here the concentric contraction was was found in both eyes, although of much less amount in the eye free from paralysis. This case was complicated by epilepsy. The man was thirty-four years old; the recurrent paralysis began at the age of five; the epilepsy first appeared at thirteen years, and followed directly a fall from a ladder, which produced temporary unconsciousness.

The course of the disease is extremely variable. In some cases the duration of the paralysis and the intervals between the attacks seem to have continued the same; in some the attacks became more, in others less severe; in some again the periods of freedom became longer, in others shorter. There is no instance of permanent relief, perhaps because the time of observation was not sufficiently prolonged. Three cases terminated in death.

Different interpretations of the cause of the affection have been given. Some have considered it a purely functional disturbance, analogous to ordinary migraine. Others have supposed a tumor or inflammatory process at the nucleus of the nerve. Others again a pachy- or lepto-meningitis, an anomaly of the vessels, or a tumor at the base.

It seems to me very difficult to admit the theory of a purely functional disturbance, especially in view of the partial persistence of the paralysis noted in several cases between the attacks. There are also difficulties in the way of an assumption of a disease-process at the nucleus, lasting for many years, extensive enough to involve all branches of the nerve, subject to frequent exacerbations, and yet never affecting any other nerves, either of the same or opposite side. A basal origin appears more probable.

The results of autopsy in the three fatal cases also point to the base as the seat of the trouble. Gubler's patient died after a short illness with delirium running into coma. A plastic exudation surrounded the right motor oculi. There were other lesions, but this was the one which seemed best to explain the paralysis.

Weiss's patient died of tuberculosis of the lungs. The right motor oculi was flattened, gray, and showed a tubercular enlargement at the point where it issued from the crus cerebri. The granulations did not extend into the brain substance.

Thomsen's case passed from his observation, and died about a year later of gangrene of the lungs. The end of the case was reported by Richter. There was a fibro-chondroma of the right motor oculi, which doubled its size, but had developed in such away as to separate the nerve fibres without destroying them.

In my own case the history points pretty distinctly to a direct connection between the aural disease and the paralysis of the third nerve. There can scarcely be a doubt that a meningitis was set up by the aural disease in the twin sister. It seems here not unreasonable to suppose an exudation or thickening over a limited portion of the dura, which makes pressure on the motor oculi whenever a re-excitation of inflammation in the ear with retention of pus induces an increased vascularity or swelling. And when it is remembered that the third nerve penetrates the dura some little distance farther forward than the fourth and sixth, it is seen that such a limited thickening might readily be situated so as to affect the third nerve alone.—*Boston Medical and Surgical Journal.*

